# Mountain caves of the central region of Veracruz: A vertebrate biodiversity reservoir in a Neotropical hotspot

**DOI:** 10.1371/journal.pone.0306105

**Published:** 2024-08-09

**Authors:** Alberto Hernández-Lozano, Arturo González-Zamora, Martha L. Baena, Yareni Perroni-Ventura, Diana Gisell Juanz-Aguirre, Israel Huesca-Domíguez

**Affiliations:** 1 Instituto de Investigaciones Biológicas, Posgrado en Biología Integrativa, Universidad Veracruzana, Xalapa, Veracruz, Mexico; 2 Unidad de Manejo para la Conservación de la Vida Silvestre La Coruja, Alberto Calderón, Xalapa, Veracruz, Mexico; 3 Instituto de Investigaciones Biológicas, Academia de Zoología, Universidad Veracruzana, Xalapa, Veracruz, Mexico; 4 Instituto de Ecología y Biotecnología Aplicada, Campus para la Cultura, las Artes y el Deporte, Universidad Veracruzana, Xalapa, Veracruz, Mexico; University of Cincinnati, UNITED STATES

## Abstract

The mountain region of central Veracruz, Mexico hosts a large system of karst and volcanic caves that are unexplored. In particular, the vertebrates that inhabit these subterranean ecosystems are unknown. This study evaluated the diversity of mammals, birds, reptiles, amphibians, and fish in three environments (euphotic, disphotic, and aphotic) of 16 caves of different geological origin (12 karst caves and 4 volcanic caves) distributed along an altitudinal gradient (300–2400 m a.s.l.). We found a richness of 242 vertebrate species (184 birds, 30 mammals, 15 reptiles, 12 amphibians, and 1 fish) and an abundance of a total of 11,323 individuals (4,969 mammals, 6,483 birds, 36 reptiles, 27 amphibians, and 5 fish). The richness of all vertebrate classes was higher in karst than in volcanic caves. Vertebrate diversity was also higher at mid-altitudes between 600–899 m a.s.l. Diversity varied between environments, where bird and reptile richness was higher in the euphotic environment, while mammal and amphibian diversity was higher in the aphotic environment. The similarity in the composition of vertebrate species does not depend on the distance between karstic and volcanic caves. Volcanic and karst caves shared on average up to 70% and 55% of vertebrate species, which indicates that only 30% and 45% of species, respectively, is different in each cave type. Given the vulnerability and fragility of these subterranean ecosystems, as well as the important diversity that they contain, we recommend including the caves of the central region of Veracruz in the conservation agenda of local governments and communities. Community-based conservation can help ensure the presence of vertebrate species in the caves of this region.

## Introduction

Karst and volcanic caves have an implicit biological value worldwide since they contain unique and complex environments and organisms such as the vertebrate species that are able to inhabit these subterranean ecosystems [1]. Cave vertebrates have been poorly studied on a global scale and studies have mainly focused on bats and fish [2]. Despite this, vertebrates of all classes (i.e., mammals, birds, reptiles, amphibians, and fish) use caves and find in them suitable habitat conditions to survive [3].

The distribution of vertebrate biodiversity in cave systems, particularly in relation to the floor and altitudinal levels inside caves, has been attempted to be explained on the basis of different theories such as resource availability [4], the theory of microclimatic gradients [5,6], the theory of trophic dynamics through food networks and trophic niches [7], and the theory of ecological adaptations and restrictions [8]. Given that they are complex systems, not all theories can be applied, and thus many biodiversity studies in caves are only descriptive.

Three environments are generally distinguished in caves: euphotic, which is characterized by variable sunlight conditions; disphotic, which is also known as the twilight zone; and aphotic, which comprises spaces in total darkness and high humidity conditions [9]. The fauna in caves is classified according to how long the organisms stay in these environments. Therefore, fauna that occurs facultatively or occasionally in these environments is known as trogloxene, the fauna that uses these sites seasonally is known as troglophile, and the fauna that completely develops inside caves is known as troglobiont [10–12].

The characteristics of the surrounding landscape may have an effect on the biodiversity of vertebrates in caves. For example, cave entrances are described as transition zones between the epigean and hypogean environments, where there is lower environmental stability because food resources are limited under twilight conditions [4]. Thus, based on the specific and unique conditions of each cave, both of karst and volcanic origin, it is possible to hypothesize about the influence of the light conditions on the capacity of vertebrates to use these environments permanently or temporally [7,13]. In these ecosystems, each cave has specific ecological limits and imposes environmental restrictions on vertebrates in such a way that only the species with a greater capacity to adapt to these extreme environments are able to use them, which can lead to endemism [14]. Therefore, even though a lower biodiversity of vertebrates has been recorded in environments related to volcanic caves, endemic species have also been found in karst caves [15].

In this study, we analyzed vertebrate communities to highlight how the abundance and diversity of native mammals, birds, amphibians, reptiles, and fish are influenced by cave origin (karst or volcanic). The use of biodiversity profiles based on Hill numbers [16] allows for a complete characterization of the biodiversity of a community [17] because they combine information on species richness, species rarity, and species dominance. We therefore used beta-diversity metrics to assess how vertebrate species composition varies between karst and volcanic caves, between altitudinal zones, and between environments inside the caves.

Considering the specific geological features of karst and volcanic caves [1] and given that biodiversity in caves is influenced by the characteristics of the surrounding landscape [4], karst caves would be expected to contain a higher biodiversity of vertebrates (mammals, birds, amphibians, reptiles, and fish) compared to volcanic caves. Moreover, according to altitudinal zonation, terrestrial biodiversity is highest at mid-altitudes, and thus the highest biodiversity of vertebrates would be expected to occur in caves located in the middle zone of the altitudinal gradient. Furthermore, because light conditions influence biodiversity in the internal environment of caves, the euphotic zone would be expected to have a higher richness of trogloxene birds and reptiles, the disphotic zone would be expected to have a lower richness of all vertebrate classes, and the aphotic zone would be expected to have a higher biodiversity of some troglobiont mammals (particularly bats) and a lower richness of amphibians and fish.

Examining beta diversity in karst and volcanic caves along an altitudinal gradient allows the identification of species turnover patterns [18]. Different caves at varying altitudes may harbor distinct species assemblages due to differences in environmental conditions [19]. This information is crucial for understanding the distribution patterns of vertebrates and identifying factors that influence species composition in karst and volcanic caves across different elevations [20].

We predicted that more abundant species such birds and mammals would show a higher β-diversity between cave types, between karst and volcanic caves at different altitudinal zones, and between environments in both cave types, whereas less abundant species such as reptiles and amphibians would have a lower β-diversity. We also predicted that the difference in vertebrate β-diversity would be primarily due to species richness rather than to species replacement [21,22]. Finally, caves in closer proximity to each other would be expected to have more similar species.

## Materials and methods

### Ethics statement

Our study was non-invasive and adhered to the legal requirements of Mexican law (NOM-059-SEMARNAT-2010). Vertebrates were sampled during field expeditions under the field permits SGPA/DGVS/10344/21 provided by the Secretaría de Medio Ambiente y Recursos Naturales of Mexico (SEMARNAT).

### Study area

The study area is located in the region of central Veracruz, at the intersection of the Sierra Madre Oriental and the Trans-Mexican volcanic belt [23]. The area exhibits high topographic heterogeneity with an elevation gradient (300 to 2,400 m a.s.l.) with two climatic areas: from warm at the lower part, to temperate at mountain mid-elevations, to cold at the higher regions [24]. The orographic and climatic differences within the area allow the presence of six vegetation types [24] from the dry environments at elevated temperatures (tropical semideciduous forest and tropical oak forest), to the humid temperate areas (humid montane forest, pine-oak forest), to the cold and dry areas at the higher parts of the gradient (pine and fir forests) [23]. In addition, from a biogeographic perspective, this area is located at the confluence between the Nearctic and Neotropical regions, and it thus has a significant biogeographic value and an even greater biodiversity value [23,24].

For this study, 12 caves of karst origin and 4 of volcanic origin were selected, comprising an area from the northeast of the Nauhcampatépetl volcano (19°29′31″N, 97°09′00″O) to the southeast of the Citlaltépetl volcano (19°02′N, 97°16′O). Modified cave topographies were used for each cave to carry out exploratory visits and adjust the methodology for vertebrate sampling.

According to altitudinal zonation, the elevation gradient was divided into four altitudinal zones: Z1 (300–599 m a.s.l), Z2 (600–899 m a.s.l), Z3 (900–1199 m a.s.l.), and Z4 (2100–2400 m a.s.l.). There was a mean difference of 200 m between the altitudinal zones. Four karst caves were located at Z1: Maguey (MY), Atoyac (AY), nacimiento de 7 aguas (NC), and ojo de agua grande (OJ). Eight karst caves were located at Z2: Pinoltepec (PN), la Reja (RE), el Aire (AI), la Palma (PL), el boquerón de Capulapa (BQ), el sótano de Tepeapulco (TP), and la cueva Pintada (PI), as well as the volcanic cave de Tenampa (TN). Two caves were located at Z3: el sótano kárstico de los Maltos (MT), of karst origin, and the volcanic cave of la Garganta (GT). Finally, the caves de la Escalera (ES) and del Volcancillo (VC), both of volcanic origin, were located at Z4 (Table 1). The cave at the highest altitude (cueva del Volcancillo) was located at 2392 m a.s.l., and the cave at the lowest altitude (cueva del Maguey) was located at 339 m a.s.l. The average distance between all caves was 43.4 km, between karst caves it was 33.3 km, and between volcanic caves it was 14.3 km.

**Table 1:** Diversity of vertebrates in karst and volcanic caves of central Veracruz, Mexico. The information in parentheses is the abundance of each group.

Origin	Zone	Cave	Alt (N)	Lat (W)	Mammals	Birds	Reptiles	Amphibians	Fish
**Karst**	Z1	MY	18.83426°	-96.72428	5 (207)	63 (474)	1 (1)	1 (5)	0 (0)
**Karst**	Z1	AY	18.92049°	-96.76640°	3 (47)	40 (217)	1 (1)	0 (0)	0 (0)
**Karst**	Z1	NC	18.76701°	-96.84332°	1 (100)	36 (10)	0 (0)	0 (0)	0 (0)
**Karst**	Z1	OJ	18.92726°	-96.87612°	1 (1)	65 (576)	0 (0)	1 (2)	0 (0)
**Karst**	Z1	PN	19.43845°	-96.74087°	5 (336)	68 (392)	4 (7)	6 (7)	0 (0)
**Karst**	Z2	RE	19.38432°	-96.78542°	8 (102)	55 (586)	1 (1)	1 (1)	0 (0)
**Karst**	Z2	AI	19.38692°	-96.78669°	4 (388)	41 (176)	1 (1)	2 (2)	0 (0)
**Karst**	Z2	PL	19.04021°	-96.83071°	4 (248)	65 (919)	1 (1)	0 (0)	1 (4)
**Karst**	Z2	BQ	19.09101°	-96.88980°	7 (3868)	25 (120)	0 (0)	0 (0)	0 (0)
**Karst**	Z2	TP	19.41863°	-96.81271°	2 (3)	37 (354)	2 (2)	1 (1)	0 (0)
**Karst**	Z2	PI	19.07030°	-96.92583°	5 (66)	26 (161)	0 (0)	2 (3)	1 (1)
**Volcanic**	Z2	TN	19.59878°	-96.86581°	2 (156)	41 (281)	3 (4)	2 (2)	0 (0)
**Karst**	Z3	MT	19.07083°	-96.92648°	2 (23)	48 (124)	0 (0)	2 (2)	0 (0)
**Volcanic**	Z3	GT	19.62227°	-96.89670°	3 (46)	21 (46)	0 (0)	1 (2)	0 (0)
**Volcanic**	Z4	ES	19.61691°	-96.88589°	4 (283)	23 (138)	2 (9)	0 (0)	0 (0)
**Volcanic**	Z4	VL	19.54176°	-96.93687°	4 (602)	42 (172)	2 (9)	0 (0)	0 (0)

Z1 = 300–599 m a.s.l., Z2 = 600–899 m a.s.l., Z3 = 900–1199 m a.s.l., and Z4 = 2100–2400 m a.s.l.

Caves: del Maguey (MY), de Atoyac (AY), del nacimiento de 7 aguas (NC), de ojo de agua grande (OJ), de Pinoltepec (PN), de la Reja (RE), del Aire (AI), de la Palma (PL), del boquerón de Capulapa (BQ), de Tepeapulco (TP), la Pintada (PI) de Tenampa (TN), de los Maltos (MT), de la Garganta (GT), de la Escalera (ES), and del Volcancillo (VL).

The average length of the volcanic caves was 1,459 m, with the longest being “la Escalera” (ES) with 3,875 m, and the shortest being “la Garganta” (GT) with 220 m. The average slope was -108 m, the lowest slope was -8 m (GT cave), and the highest slope was -215 m (ES cave). In the case of the karst caves, the average length was 851 m, with the shortest being the “Tepeapulco” (TP) cave with 42 m, and the longest being the “Palma” (PL) cave with 3,353 m. The average slope was -37 m, the lowest slope was -7 m (PI cave), and the highest slope was -98 m (TP cave).

### Environments

According to the availability of light, the environments of the caves were classified as euphotic (light entrance zone), which is exposed to the external environment, disphotic (twilight zone), which receives limited sunlight during a few hours of the day, and aphotic (deep cave zone), which is characterized by the complete absence of light [25]. Two vertebrate surveys were carried out in each cave, one in spring and one in fall of 2021.

### Vertebrate surveys and sampling completeness

For the survey of birds in the euphotic zone, two sites were established for fixed-radius (20 m) point counts [26]. The first one was located between 20–50 m outside of the main access of each site, and the second one at an approximate distance of 100–300 m from the first counting point outwards. The sampling time was of approximately 60–120 min during daytime hours (07:00–11:00) and 60–120 min during evening hours (17:00–21:00). Observations were made with a Vortex Diamondback 20-60x80 spotting scope and Vortex Diamondback HD 8x42 binoculars. Bird calls were also recorded using a Tascam Dr-05x audio recorder. Subsequently, sampling inside the caves was carried out by searching for birds on ceilings and within crevices in established sites using Fenix HM65R-T headlamps [21].

For mammals, reptiles, amphibians, and fish, timed searches of 20 min were performed within a 2 m radius in established sites in the euphotic, disphotic, and aphotic environments [21,27]. For mammals, direct observations and the presence of tracks and traces [28] were also recorded, and identifications were made using taxonomic guides [29]. For amphibians and reptiles, photographic records were obtained for their subsequent identification using herpetological guides [30–32]. The guides by Howell and Webb [33] and Sibley [34] were used for the identification of birds and the guide by Miller [35] was used for the identification of fish.

### Statistical analysis

The richness and abundance of each vertebrate species were pooled per cave origin, altitudinal zone, and environment. We estimated species richness in each site using Hill numbers, which allow for comparisons between species richness estimates allotting more weight to rare (q = 0), common (q = 1), dominant (q = 2), and very dominant (q = 3) species [36]. Vertebrate assemblages in karst and volcanic caves were characterized by their diversity and composition using alpha-diversity and beta-diversity. Analyses were conducted for each of the following vertebrate groups: mammals, birds, reptiles, and amphibians; fish were excluded from the analysis due to their low representativeness. Statistical analyses were run using the R analysis language [37].

**Fig 1:**
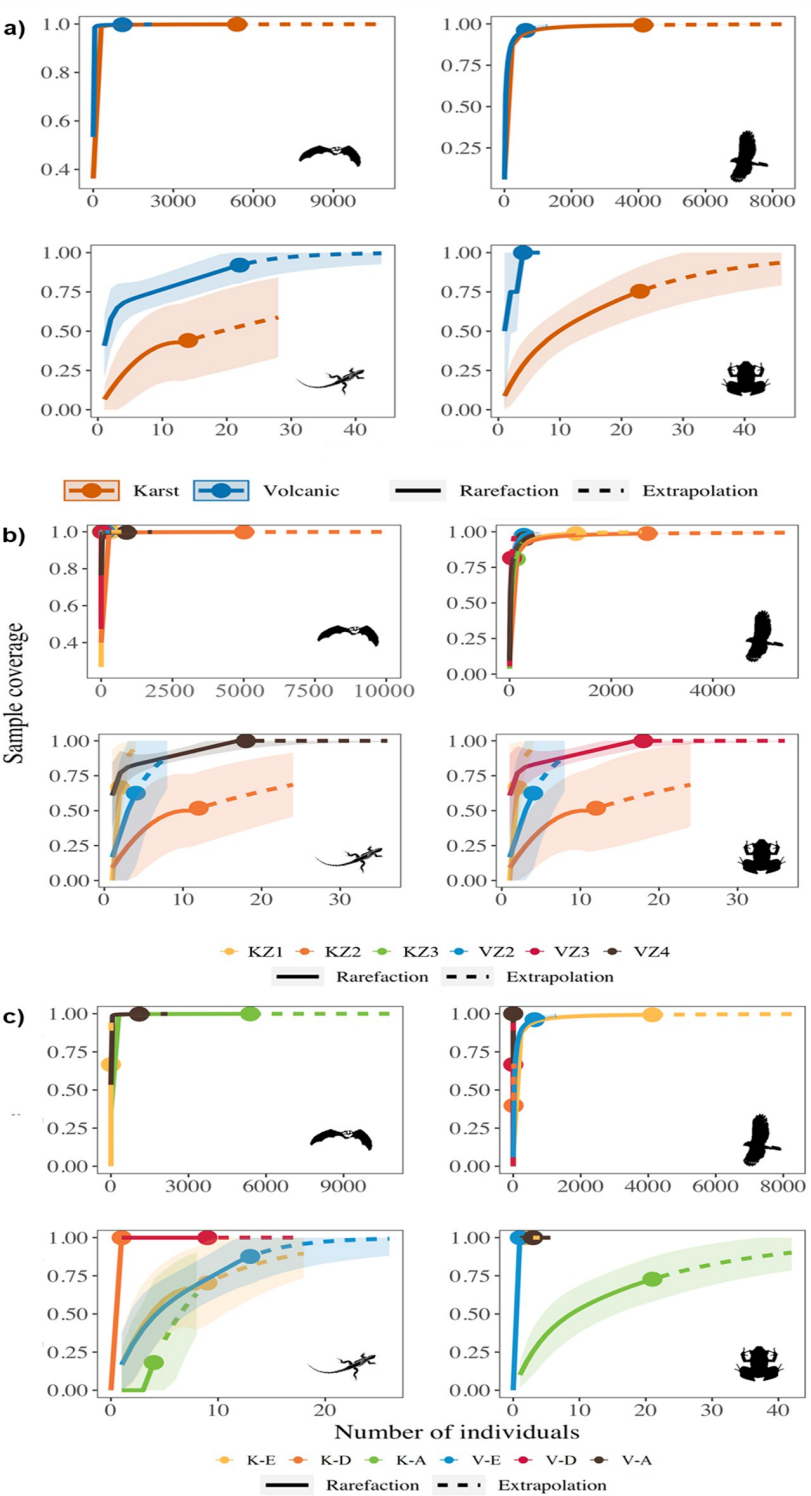
Sample coverage of vertebrate classes according to cave origin (a), altitudinal zone (b), and cave environment (c).

In order to evaluate the completeness of the sampling process, extrapolation and interpolation procedures were carried out with 100 randomizations using the iNEXT software version 1.3.0 available online at https://chao.shinyapps.io/iNEXTOnline ([38]. The richness values of each vertebrate class were statistically compared between cave origins, altitudinal zones, and environments using 95% confidence intervals. Sampling completeness was variable in karst and volcanic caves, but most groups reached between 70–100% sampling completeness (Fig 1).

To determine the similarity in species richness between karst and volcanic caves, a cluster analysis with UPGMA clustering method was conducted in PAST 4.03, using Bray-Curtis distance matrix and log (x + 1) transformation. To evaluate the relationship between the geographical distance of karstic and volcanic caves and vertebrate composition (Bray-Curtis dissimilarities), the Mantel test [39] with 9,999 permutations was performed using the vegan R package [40].

We computed β-diversity on abundance data using the Jaccard dissimilarity coefficient. To capture the processes underlying species dissimilarity among communities, we partitioned the overall β-diversity values into their species replacement (change in biodiversity due to species turnover) and species richness (change in biodiversity due to differences in species richness) components [41]. We calculated β-diversity between karst and volcanic caves, between caves at different altitudinal zones, and between cave environments using the function “beta.div” in the R package “adespatial” version 0.3–14 [42].

Beta-diversity decomposition analysis. To evaluate the variation in the composition of vertebrate species assemblages within and between caves, between altitudinal zones, and between cave environments, we performed a beta-diversity partitioning analysis considering that:

βtotal=βrepl+βrich

where βtotal represents the total variation in vertebrate species composition between assemblages, βrepl accounts for the variation due to species replacement, while βrich refers to the absolute differences in species richness [41,43].

## Results

We recorded 11,520 individuals (4,969 mammals, 6,483 birds, 36 reptiles, 27 amphibians, and 5 fish) belonging to 78 families, 187 genera, and 242 vertebrate species (184 birds, 30 mammals, 15 reptiles, 12 amphibians, and 1 fish) in the 16 caves sampled (Tables 1 and S1). Birds showed the highest number of species in karst and volcanic caves (153 and 89, respectively), while amphibians and fish had the lowest number of species. Most vertebrates were birds (57.25%) and mammals (43.87%), which together represented 99.4% of all the vertebrates recorded (S1 Table).

### Species diversity (Hill numbers)

**Fig 2:**
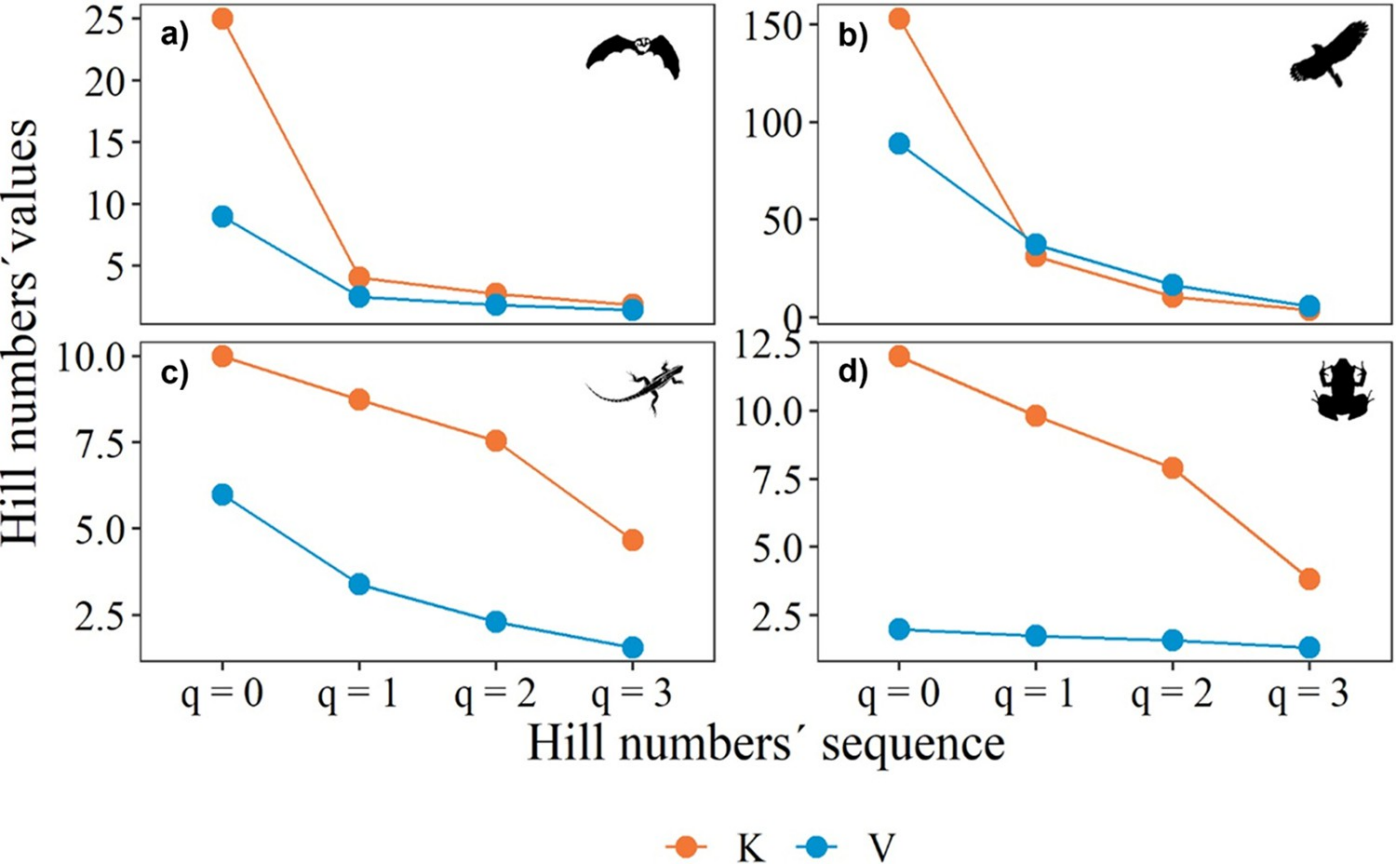
Diversity (Hill numbers) profiles of mammals (a), birds (b), reptiles (c), and amphibians (d) in karst (K) and volcanic (V) caves.

The Hill number profiles showed contrasting patterns between karst and volcanic caves for all vertebrate classes. The highest species richness (q^0^) of all vertebrates sampled was observed in karst caves, with birds and mammals being the classes with the highest species richness (Fig 2A and 2B). However, only reptiles and amphibians had a higher richness of common, dominant, and very dominant species in karst caves (Fig 2C and 2D). In contrast, volcanic caves had the lowest richness (q^0^) and diversity of vertebrates (q^0,1,2,3^).

**Fig 3:**
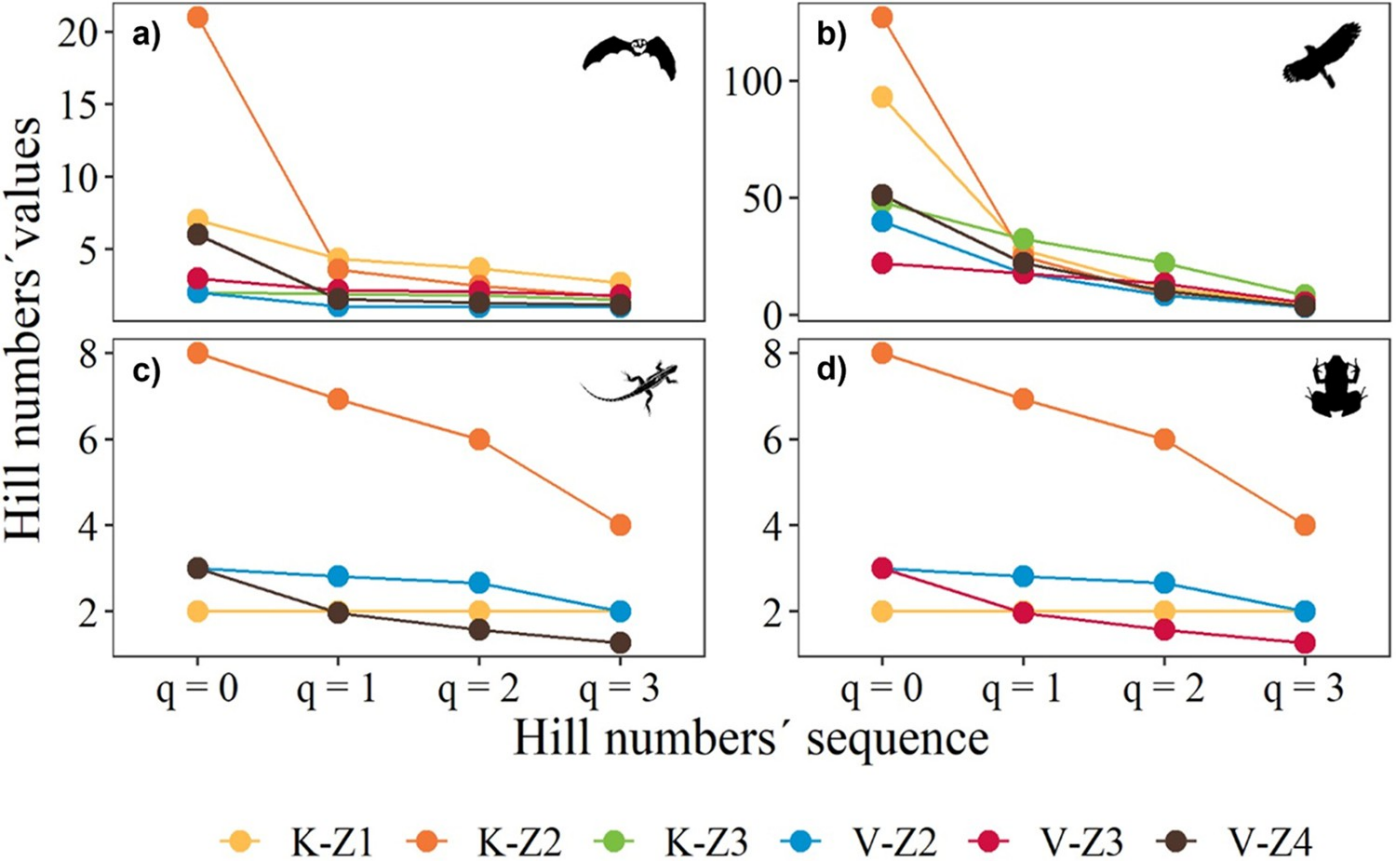
Diversity (Hill numbers) profiles of mammals (a), birds (b), reptiles (c), and amphibians (d) in karst and volcanic caves at different altitudinal zones: Z1 (300–599 m a.s.l), Z2 (600–899 m a.s.l), Z3 (900–1199 m a.s.l.), and Z4 (2100–2400 m a.s.l.).

The analysis of karst and volcanic caves at different altitudinal zones also showed contrasting patterns. In terms of species richness (q^0^), birds were the class with the highest richness in the karst caves located in Z1 (300–599 m a.s.l.) and Z2 (600–899 m a.s.l.). Mammals and the rest of the vertebrates had a relatively high richness, especially in karst caves at Z2. Although reptiles and amphibians had fewer species than the other vertebrate classes, they showed more common species (q^1^), dominant species (q^2^), and very dominant species (q^3^) in karst caves at Z2. In contrast, volcanic caves showed the lowest diversity of the vertebrates sampled (Fig 3).

**Fig 4:**
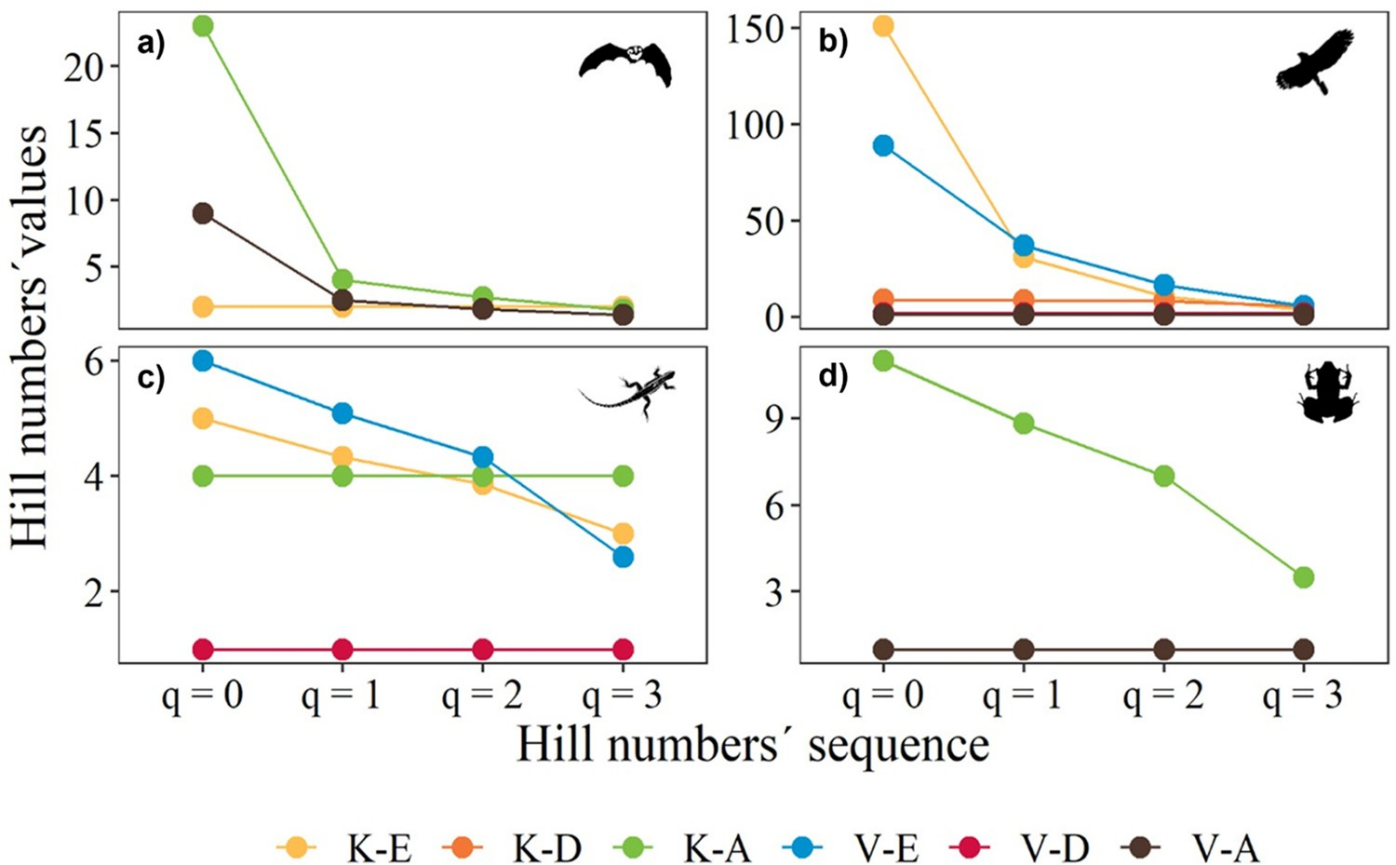
Diversity (Hill numbers) profiles of mammals (a), birds (b), reptiles (c), and amphibians (d) in the euphotic, disphotic, and aphotic environments of karst (K-E, K-D, K-A) and volcanic (V-E, V-D, V-A) caves.

**Fig 5:**
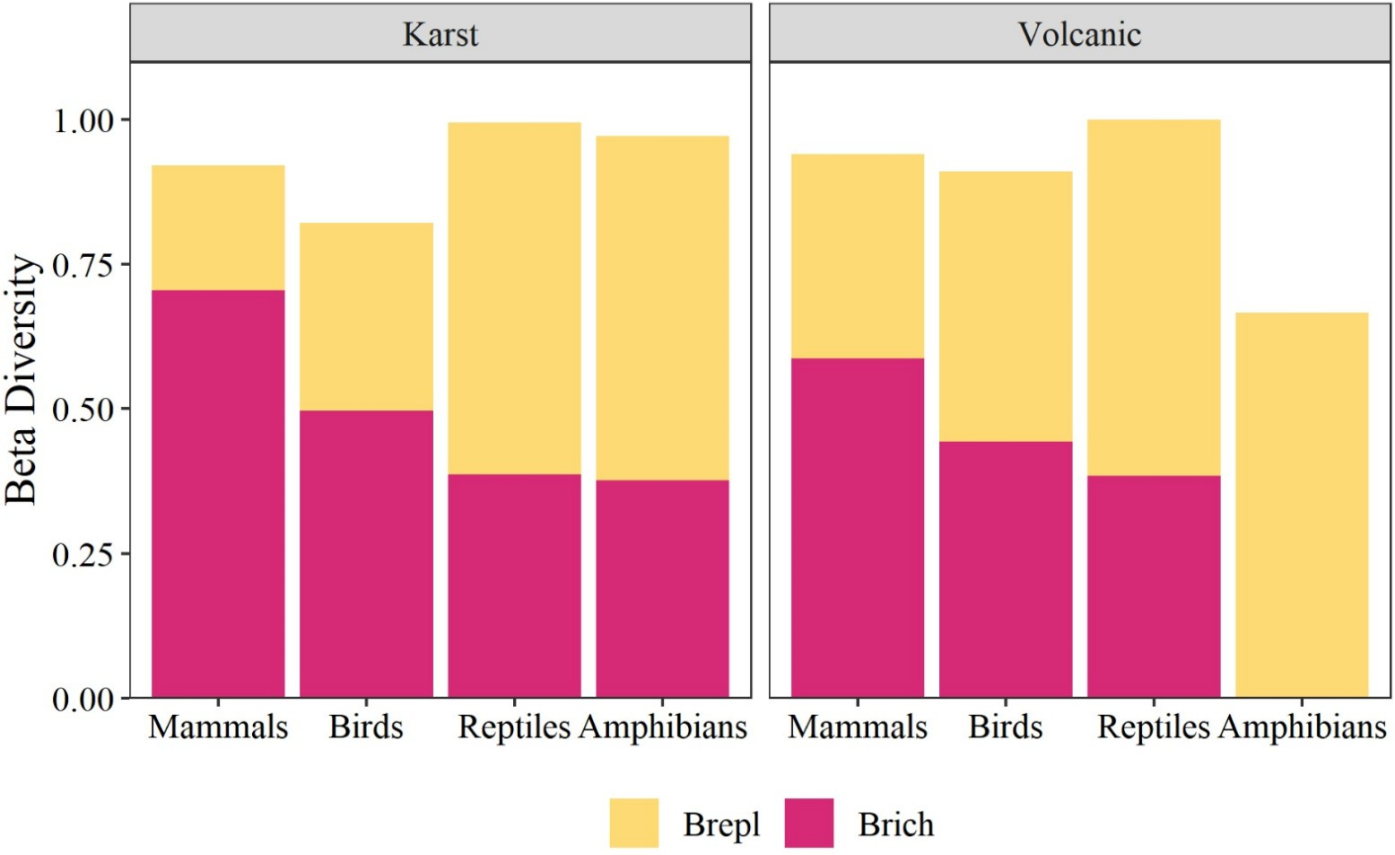
β-diversity values of four vertebrate classes calculated between karst and volcanic caves. The total height represents the overall β-diversity. Overall β-diversity is partitioned into replacement (repl. in yellow) and richness (rich in red) components.

The Hill number profiles according to cave environment showed the highest bird species richness (q^0^) in the euphotic environment of karst and volcanic caves, but this environment also showed a lower number of common species (q^1^), dominant species (q^2^), and very dominant species (q^3^) of this vertebrate class (Fig 5). Mammals and amphibians in the aphotic environment of karst caves showed the same pattern. Reptiles (with fewer species than the other vertebrate classes) showed high species richness (q^0^) but lower q^1^, q^2^, and q^3^ in the euphotic environment of volcanic and karst caves. In contrast, the three environments of volcanic caves showed low and more constant numbers of species, common, dominant, and very dominant species of all vertebrate classes (Fig 4).

### Beta diversity in Karst and volcanic caves

The β-diversity values in karst caves were higher for mammals and birds and were due to differences in species richness (70% and 50%, respectively). In contrast, the β-diversity values obtained for reptiles and amphibians were similar and were mostly due to differences in species replacement (60% and 58%, respectively) (Fig 5).

In volcanic caves, mammals exhibited the highest β-diversity values, which were due to differences in species richness (65%). In the case of birds, the β-diversity values were similar and were due to differences in species richness (45%) and replacement (55%). With respect to amphibians and reptiles, species replacement contributed more substantially to the β-diversity values of these groups (100% and 60%, respectively) (Fig 5).

### Beta diversity in caves between altitudinal zones

**Fig 6:**
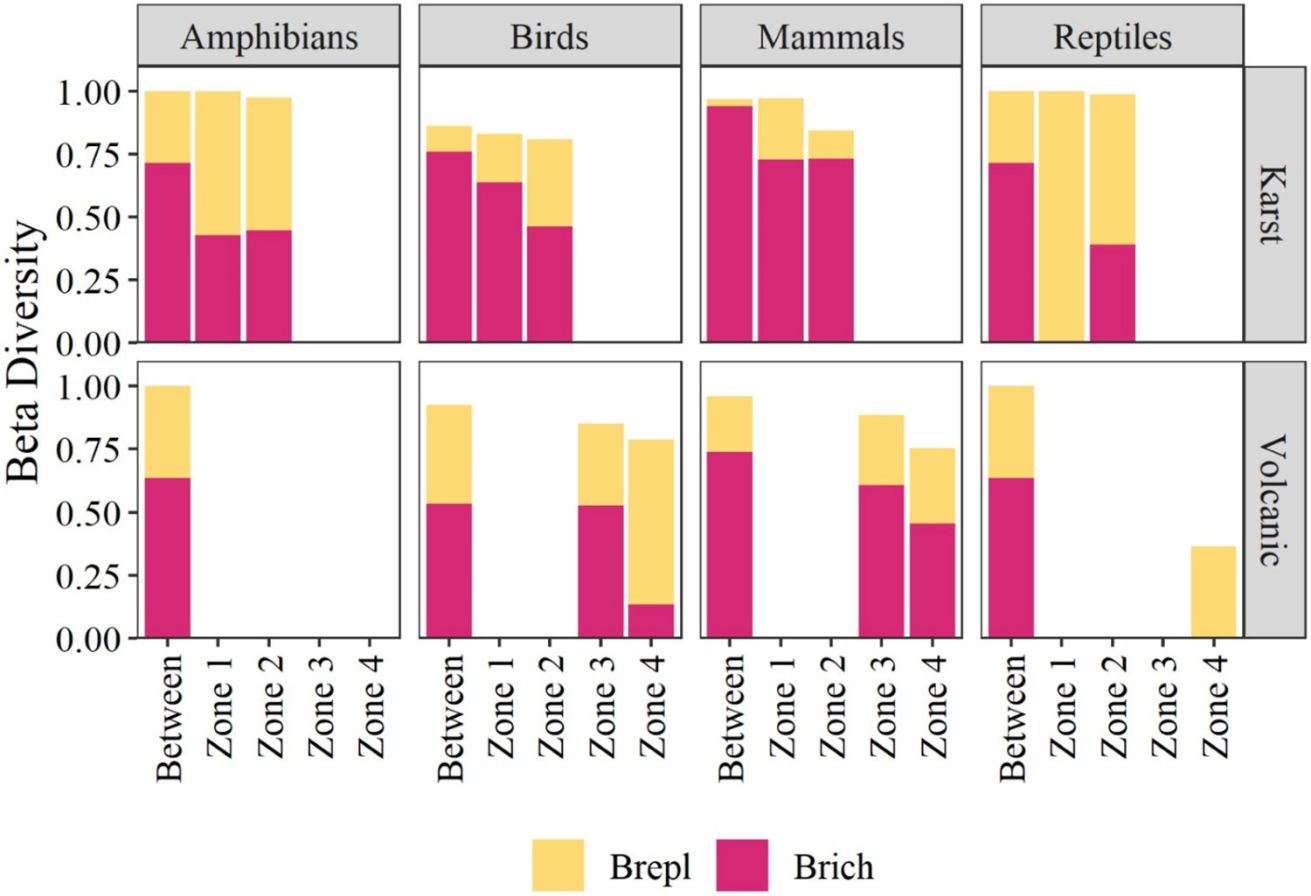
β-diversity values of four vertebrate classes calculated between altitudinal zones. The total height represents the overall β-diversity. Overall β-diversity is partitioned into replacement (repl. In yellow) and richness (rich in red) components.

In karst caves, mammals and birds were the vertebrate classes with the highest β-diversity between caves but also between altitudinal zones Z1 and Z2 (Fig 6). The β-diversity values of amphibians were similarly influenced by differences in species richness and species replacement. In the case of reptiles, species replacement contributed more substantially to the β-diversity of this group, especially in Z1. The vertebrates in karst caves at Z3 and Z4 did not show β-diversity values (Fig 6). In volcanic caves, species richness dominated the β-diversity of all vertebrate classes, especially in Z3 and Z4 (Fig 6).

### Beta diversity in cave environments

**Fig 7:**
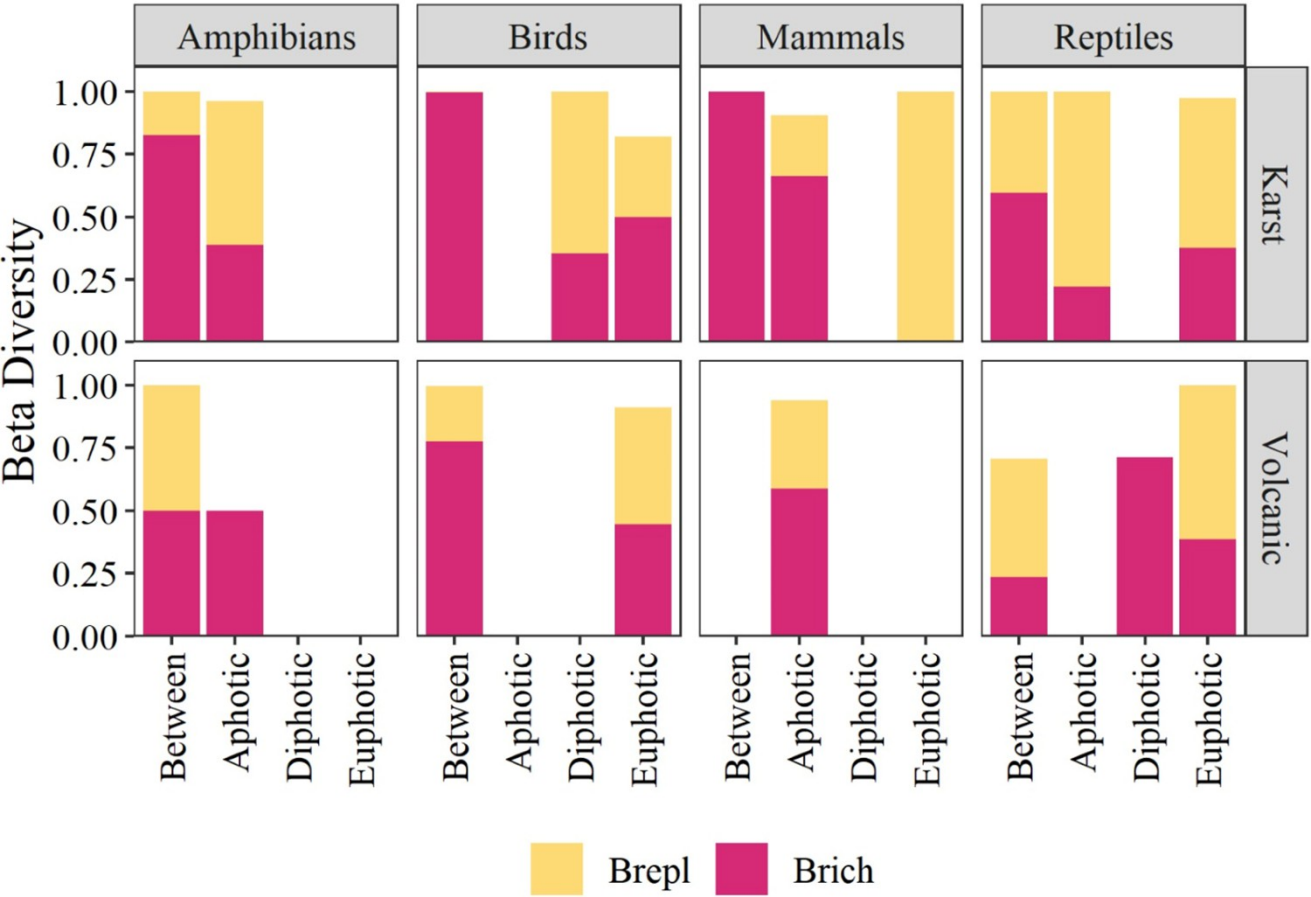
β-diversity values of four vertebrate classes calculated between the environments of karst and volcanic caves. The total height represents the overall β-diversity. Overall β-diversity is partitioned into replacement (repl. In yellow) and richness (rich in red) components.

In the aphotic environment of karst caves, species richness showed the highest contribution to the β-diversity of mammals and amphibians. In the disphotic environment, differences in species replacement (60%) and richness (40%) influenced the β-diversity of birds. Finally, in the euphotic environment, birds and reptiles showed similar β-diversity values, which were due to both species replacement and richness, and species replacement contributed 100% to the β-diversity values of mammals (Fig 7).

In volcanic caves, species richness contributed in a higher proportion to the β-diversity of mammals and birds in the aphotic and euphotic environments. Species richness also contributed in a higher proportion to the β-diversity of amphibians and reptiles but in the aphotic and disphotic environments (Fig 7).

### Similarity in species richness

**Fig 8:**
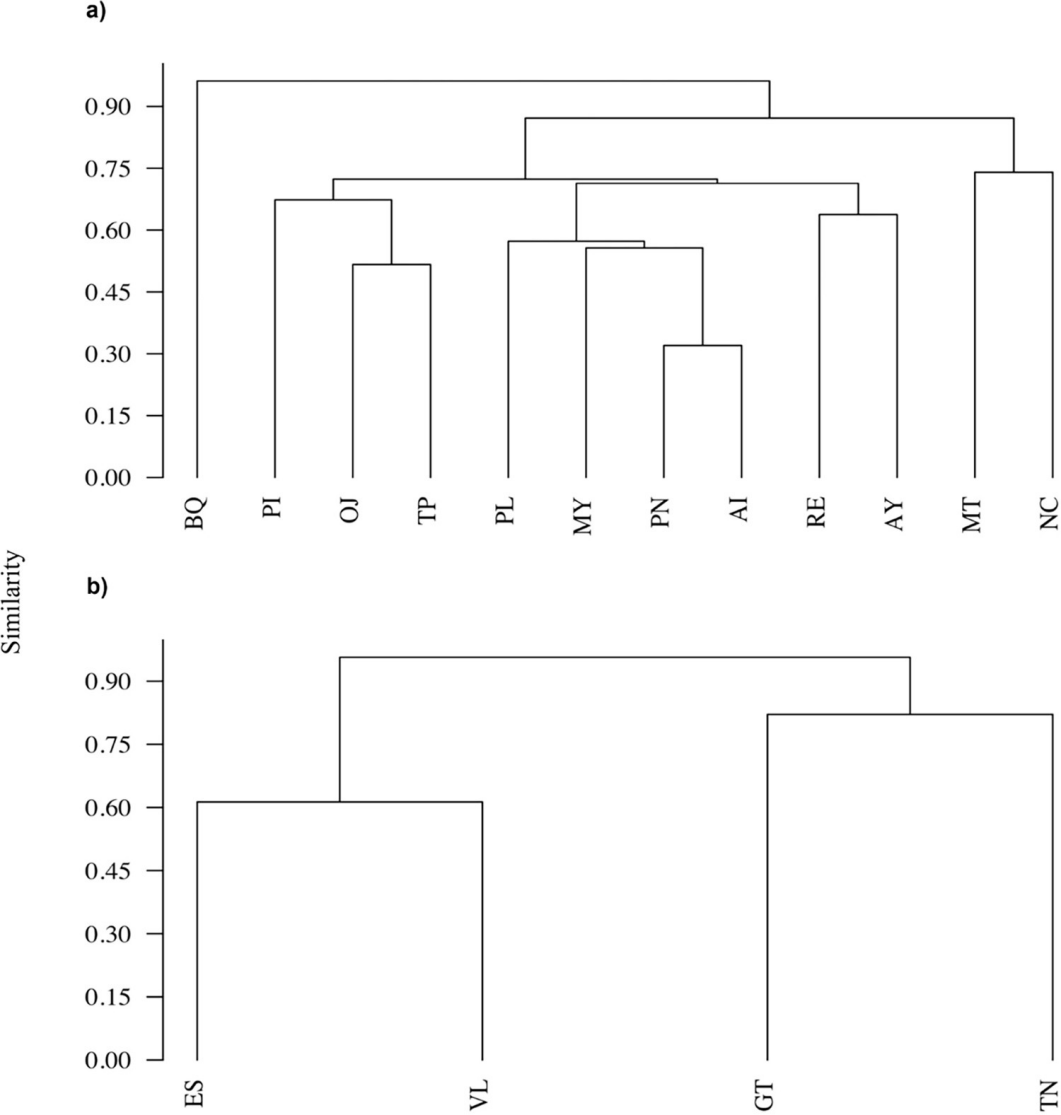
Dendrogram of similarity in vertebrate species richness between caves of karst (A) and volcanic (B) origin (Bray Curtis index—cophenetic correlation 0.89 and 0.99, respectively) in Veracruz, Mexico. Karst caves: MT = cueva de los Maltos, NC = cueva del nacimiento de 7 aguas, MY = cueva del Maguey, PN = cueva de Pinoltepec, AY = cueva de Atoyac, AI = cueva del Aire, PL = cueva de la Palma, RE = cueva de la Reja, TP = cueva de Tepeapulco, PI = cueva la Pintada, OJ = cueva de ojo de agua grande, and BQ = cueva del boquerón de Capulapa. Volcanic caves: ES = cueva de la Escalera, GT = cueva de la Garganta, VL = cueva el Volcancillo, TN = cueva de Tenampa.

The similarity in species richness between caves showed that karst caves shared a mean of 55% of species, whereas volcanic caves shared a mean of 70% of species. The greatest similarity was found between the karst caves MT and NC, which shared up to 75% of the total species, followed by the karst caves PI, OJ and TP, which shared 70% of the total species. The volcanic caves ES and VL shared 807% of species richness (Fig 8). The Mantel test showed that similarity in species richness is directly related to the distance between karts (r = 0.15, p = 0.11) and volcanic (r = 0.42, p = 0.33) caves.

## Discussion

The land region of central Veracruz is considered a biodiversity hotspot, but its cave diversity and ecosystems are underexplored. We found a high vertebrate diversity (i.e., 184 bird species, 30 mammal species, 15 reptile species, 12 amphibian species, and 1 fish species) in karst and volcanic caves. To our knowledge, this is the first study focusing on the diversity of all vertebrate classes in karst and volcanic caves along an altitudinal gradient in Eastern Mexico. Thus, the knowledge generated in this study contributes to filling the information gap on the diversity of vertebrates that inhabit the subterranean ecosystems of this region and can help to design conservation actions for vertebrate diversity and cave ecosystems.

### Effective number of species in caves

We hypothesized that karst caves would contain a higher diversity of individuals of all vertebrate classes than volcanic caves. Vertebrate species showed a clear pattern in both cave types: high species richness and a few common, dominant, and very dominant species. These results agree with different studies carried out in China [3], Colombia [44], and the south of Veracruz (Mexico), where the presence (but not diversity) of all vertebrate classes has been recorded [45,46]. Therefore, our results confirm that karst caves provide natural habitats for all vertebrate classes and play a significant role in the maintenance of cave vertebrate diversity.

The effective number of species in karst caves indicates that bird and mammal species have a higher richness (q^0^) but lower evenness due to the high presence of common (q^1^), dominant (q^2^), and very dominant species (q^3^). Reptiles and amphibians showed a relatively similar pattern. Conversely, in volcanic caves, all vertebrate classes showed high evenness due to more constant effective species numbers for all q orders. Populations of vertebrates such as birds and mammals tend to have more species and fewer common and dominant species [47], and therefore the Hill number profiles observed in our study are consistent with what has been reported in terrestrial ecosystems for these groups of vertebrates [9,48]). Hence, a higher species richness (q^0^) and fewer common (q^1^), dominant (q^2^) and very dominant species (q^3^) of vertebrates is the rule in caves and not the exception. This indicates the capacity of these vertebrate groups to inhabit cave environments, even with the habitat restrictions encountered in them. Despite the environmental limitations of light, temperature, and humidity in the caves, we found a high abundance, species richness, and diversity of vertebrates. This indicates that vertebrates can adapt to these conditions, perhaps because they find resources such as food and suitable spaces.

### Effective number of species in caves at different altitudinal zones

Many species of vertebrates were observed in karst and volcanic caves across altitudinal zones. Our findings indicate that the species diversity of almost all vertebrate classes was higher in karst caves at mid-altitudes (600–899 m a.s.l.). According to altitudinal zonation, terrestrial diversity is highest at mid-altitudes [49]. Mid-altitudes are characterized by a greater variety of forests (i.e, cloud forest, oak forest, and dry forest), which decreases at higher and lower altitudes [49]. In fact, there is a variety of habitats in this region [24]. This variety of forests that composes the altitudinal landscape and surrounds caves [50] can serve as habitats for vertebrates and promote local diversity [51,52].

The effective number of species in karst caves at mid-altitudes indicated a higher species richness (q^0^) of birds and mammals, whereas reptiles were the class with the lowest species richness. In volcanic caves, all vertebrate classes showed high species evenness due to more constant effective species numbers for all orders of q (^0,1,2,3^). The communities with the lowest richness were those at the highest (Z4) and lowest (Z1) altitudinal zones. Caves located at mid-altitudes may offer more stable environmental conditions that promote the presence of vertebrate species [53] such as birds and mammals, as we found in our study. In addition to environmental conditions, caves located at mid-altitudes may offer habitat resources such as nesting sites for bird communities and shelter for mammals, particularly bats, as has been documented in other cave systems along altitudinal gradients [54].

### Effective number of species in cave environments

Vertebrate diversity in caves is used as an indicator of habitat quality for troglobitic, trogloxenic, and troglophilic vertebrates [1], and the distribution of vertebrates inside karst caves seems to depend on several factors such as the size and distribution of entrance spaces, organism mobility, and food distribution [55]. Our results indicate that, regardless of the environment occupied by vertebrates, all groups show the same pattern of higher species richness (q^0^) and a few common (q^1^), dominant (q^2^), and very dominant species (q^3^). Our findings indicate that birds and reptiles in the euphotic environment of karst and volcanic caves, mammals in the aphotic environment of both cave types, and amphibians in the aphotic environment of karst caves showed a relatively similar pattern of effective number of species. Mammals showed a similar pattern in the aphotic environment of karst and volcanic caves due to the presence of bats such as *Artibeus jamaicensis* (Chiroptera:Phyllostomidae), *Balantiopteryx io* (Chiroptera:Emballonuridae), *Myotis keaysi* (Chiroptera:Vespertilionidae), or *Corynorhinus mexicanus* (Chiroptera:Vespertilionidae). The disphotic environments of karst and volcanic caves showed the lowest number of species for all orders of q (^0,1,2,3^).

Most mammals were recorded in the aphotic environment, birds and reptiles in the euphotic and disphotic environments, and amphibians in the disphotic and aphotic environments. The euphotic environment is a transition zone between surface and subsurface environments, where there is a distinct gradient of several abiotic factors, especially light, temperature, and humidity [56]. These conditions may be important for vertebrates such as birds and reptiles, which presented the highest biodiversity and abundance in the euphotic environment. We found that the innermost environment in the caves (aphotic environment) was also attractive to vertebrates such as bats and amphibian species. This indicates that the high richness of these species responds to different resources in the aphotic environment such as shelter, microclimatic conditions, food, and water [57,58].

### Beta diversity between cave origins, altitudinal zones, and cave environments

We hypothesized that bird and mammal species would show a higher β-diversity between karst and volcanic caves along the altitudinal zones, while reptile and amphibian species would show a lower β-diversity. We also predicted that the difference in vertebrate β-diversity between environments would be primarily due to species richness rather than to species replacement. Our results met these expectations, confirming thus that bird and mammal species composition is similar among caves at different altitudinal zones [59].

Contrary to what is reported in the literature [58], vertebrate beta diversity is high and is mainly due to species richness rather than species replacement. In this sense, on average, up to 50% of bird and mammal species are shared between caves. Numerous studies suggest that vertebrate species in caves are unique, which increases β-diversity in caves at a landscape scale [60]. However, we know that small disturbances in habitats surrounding caves can increase the number of vertebrate species, particularly those of birds and mammals that occupy karst and volcanic caves and their environments [61]. These vertebrate species are affected by the conditions imposed by disturbances in the external habitat and, hence, a proportion of these vertebrate species find refuge in caves [62]. Therefore, our results suggest that disturbances in external habitats surrounding caves can reduce beta diversity, increasing β-richness and decreasing β-replacement, which leads to a reduction in regional vertebrate β-diversity in caves.

Patterns of β-diversity in caves along an altitudinal gradient are the reflection of the discontinuous nature of cave ecosystems [63], and the diversity of vertebrates within the caves can be related to the distribution and displacement capacity of vertebrate species [64]. The high β-diversity values observed for all vertebrate classes confirm the presence of birds, mammals, reptiles, and amphibians in different karst and volcanic caves along the altitudinal gradient studied. These results suggest that an important proportion of similar bird and mammal species use caves as habitats along this altitudinal gradient. Birds and mammals have a high displacement capacity, which can increase the chance of occupying caves as habitats at different altitudinal zones, unlike reptiles and amphibians [64], which may have wide distribution ranges but have a lower cursorial capacity, and therefore only a few species of reptiles and amphibians are able to inhabit caves [47]. The vertebrate diversity observed in karst and volcanic caves contributes to maintaining the regional patterns of vertebrate β-diversity in the caves along this altitudinal gradient, and all vertebrate classes that inhabit caves play an important role in the local composition of cave biodiversity.

Estimations of β-diversity inside caves are essential for assessing the degree of species composition between different cave environments [65,66]. In both cave types, each chamber or passage may have unique cave characteristics [1] and environmental conditions (humidity and temperature [55]) that favor the presence of different species in different cave environments. The β-diversity observed in the different environments of karst and volcanic caves indicates that species replacement and richness contributed in a similar proportion to the β-diversity of all vertebrate classes. The high β-diversity values observed for all vertebrate classes in the three environments of karst and volcanic caves confirms the higher richness of trogloxene birds and reptiles in the euphotic environment and of troglobiont mammals and amphibians in the aphotic environment.

### Implications for conservation

Biodiversity in tropical subterranean habitats varies from place to place [67,68] and, as we found in this study, species turnover is high in karst and volcanic caves [12]. As expected, there was a relationship between species richness and distance between caves. For example, 37% of the total number of species was similar between karst and volcanic caves in close proximity to each other. These results are relevant because they also show that 63% of species are unique to each cave. Thus, regardless of the relative proximity between caves, each one contains a high percentage of unique species [1,3], especially in the case of reptiles and amphibians. Birds and mammals were the groups that shared the most species between karst and volcanic caves. The mountain region of central Veracruz, Mexico hosts a large system of karst and volcanic caves that are unexplored. If we consider the number of unexplored caves present in this region (approximately 53), the vertebrate diversity found in this study is likely to represent a fraction of the total diversity that these subterranean ecosystems can contain. Future studies should focus on assessing vertebrate diversity in other unexplored caves of the region.

Human disturbances, such as land-use change, directly affect subterranean biodiversity [69–71]. Many vertebrates that inhabit caves are especially adapted to these environments and may be highly vulnerable to human disturbances [67], particularly those that cannot move large distances such reptiles and amphibians. For example, the loss of vegetation may result in environmental degradation, especially of small, shallow caves. In this context, in the last twenty years, the conversion of forests into cattle pastures and agricultural fields has been the main land use pressure on the natural forests of central Veracruz [24], which affects the forests around caves. Considering these threats, it is becoming increasingly important to carry out conservation actions to protect subterranean environments [71,72], since the species that inhabit them, many of which are endemic, are vulnerable to human disturbances.

Protecting cave ecosystems and the vertebrates that rely on them is essential for preserving biodiversity and preventing the loss of unique species and ecological functions [73]. Our results confirm the presence of vertebrates native to Mexico listed in some risk category in the Official Mexican Standard NOM-059-SEMARNAT-2010. Four amphibians, five reptiles, 22 birds, and one mammal are found in special protection categories, two mammals, nine birds, two reptiles, and one amphibian are listed as threatened, and one bird and one mammal are listed as endangered. Conservation efforts aimed at preserving these cave ecosystems are essential for the protection of vertebrate biodiversity. We suggest including the caves of the central region of Veracruz in the conservation agenda of local governments and communities. Community-based conservation can help ensure the presence of vertebrate species in the caves of this region.

Given that this system of karst and volcanic caves is located in the transition belt between the Nearctic and Neotropical regions, it may favor a greater diversity of vertebrates and other life forms, especially of poorly studied groups such as invertebrates. Many cave ecosystems support diverse communities of invertebrates, such as cave-adapted insects and crustaceans [68]. These invertebrates serve as a food source for vertebrates, contributing to the overall biodiversity within caves [68]. Future studies focusing on the evaluation of invertebrate diversity can provide complementary information about the importance of these organisms for the ecological functions and maintenance of vertebrate food webs in the caves of this region.

## Supporting information

S1 Table Vertebrate species recorded in karst and volcanic caves at different altitudinal zone of the central region of Veracruz, Mexico. (DOCX)
