# Supplementary material for: Mountain caves of the central region of Veracruz: A vertebrate biodiversity reservoir in a Neotropical hotspot
Source: PLoS One. 2024 Aug 9;19(8):e0306105. doi: 10.1371/journal.pone.0306105 (PMC11315317; doi:10.1371/journal.pone.0306105)
Supplement: S1 Table — (DOCX) [file pone.0306105.s001.docx]

**Table. Vertebrate species recorded in karst and volcanic caves at different altitudinal zone of the central region of Veracruz, Mexico**

| **Class** | **Specie** | **Z1** | | | | **Z2** | | | | | | | | **Z3** | | **Z4** | |
| --- | --- | --- | --- | --- | --- | --- | --- | --- | --- | --- | --- | --- | --- | --- | --- | --- | --- |
|  |  | **OJ** | **NC** | **AY** | **MY** | **TN** | **PI** | **TP** | **BQ** | **PL** | **AI** | **RE** | **PN** | **GT** | **MT** | **VL** | **ES** |
| Actinopterygii | 1. *Rhamdia* sp. |  |  |  |  |  | 1 |  |  | 6 |  |  |  |  |  |  |  |
|  | 1. Sin ID | 6 | 7 |  | 2 |  | 2 |  | 1 | 7 |  | 20 | 22 |  | 1 |  |  |
| Amphibia | 1. *Charadrahyla taeniopus* |  |  |  |  |  |  |  |  |  |  |  | 1 |  |  |  |  |
|  | 1. *Eleutherodactylus cystignathoides* |  |  |  |  | 2 |  | 1 |  |  |  |  | 1 | 2 |  |  |  |
|  | 1. *Engystomops* *pustulosus* |  |  |  |  |  |  |  |  |  |  |  | 1 |  |  |  |  |
|  | 1. *Incilius valliceps* |  |  |  |  |  |  |  |  |  | 1 |  | 1 |  |  |  |  |
|  | 1. *Lithobates berlandieri* |  |  |  |  |  |  |  |  |  | 1 |  |  |  |  |  |  |
|  | 1. *Lithobates vaillanti* | 2 |  |  |  |  |  |  |  |  |  |  |  |  |  |  |  |
|  | 1. *Rheohyla miotympanum* |  |  |  | 5 |  |  |  |  |  |  |  | 1 |  |  |  |  |
|  | 1. *Rinella horribilis* |  |  |  |  |  | 1 |  |  |  |  |  | 1 |  | 2 |  |  |
|  | 1. *Smilisca* *baudinii* |  |  |  |  |  |  |  |  |  |  |  |  |  | 1 |  |  |
|  | 1. *Smilisca* sp. |  |  |  |  |  | 2 | 3 | 3 |  | 3 | 12 | 8 |  |  |  |  |
| Reptilia | 1. *Aspidoscelis* *deppii* |  |  |  |  |  |  |  |  |  | 1 |  | 2 |  |  |  |  |
|  | 1. *Aspidoscelis* *guttatus* |  |  |  |  |  |  |  |  |  |  |  | 3 |  |  |  |  |
|  | 1. *Basiliscus* *vittatus* |  |  |  |  | 1 |  |  |  |  |  |  |  |  |  |  |  |
|  | 1. *Coleonyx* *elegans* |  |  | 1 |  |  |  | 1 |  |  |  |  |  |  |  |  |  |
|  | 1. *Ctenosaura* *acanthura* |  |  |  |  | 2 |  |  |  |  |  |  |  |  |  |  |  |
|  | 1. *Kinosternon* *herrerai* |  |  |  |  |  |  |  |  |  | 1 |  |  |  |  |  |  |
|  | 1. *Kinosternon* *scorpioides* |  |  |  |  |  |  |  |  |  |  | 1 |  |  |  |  |  |
|  | 1. *Mastigodryas* *melanolomus* |  |  |  |  |  |  |  |  |  |  |  |  |  | 1 |  |  |
|  | 1. *Ninia diademata* |  |  |  |  |  |  |  |  | 1 |  |  |  |  |  |  |  |
|  | 1. *Sceloporus formosus* |  |  |  |  |  |  |  |  |  |  |  |  |  |  | 2 |  |
|  | 1. *Sceloporus mucronatus* |  |  |  |  |  |  |  |  |  |  |  |  |  |  | 7 | 7 |
|  | 1. *Sceloporus variabilis* |  |  |  |  | 2 |  |  |  |  |  |  | 1 |  |  |  |  |
|  | 1. *Scincella gemmingeri* |  |  |  |  | 3 |  |  |  |  |  |  |  |  |  |  |  |
|  | 1. *Thamnophis scalaris* |  |  |  |  |  |  |  |  |  |  |  |  |  |  | 1 |  |
|  | 1. *Tropidodipsas sartorii* |  |  |  |  |  |  | 1 |  |  |  |  |  |  |  |  |  |
|  | 1. *Sin ID* |  |  |  |  | 1 |  |  |  |  |  |  | 1 |  |  | 1 | 2 |
| Birds | 1. *Actitis macularius* |  |  |  | 5 |  | 3 |  |  |  |  |  | 1 |  |  |  |  |
|  | 1. *Aegolius acadicus* |  |  |  |  |  |  |  |  |  |  |  |  |  |  |  | 3 |
|  | 1. *Agelaius phoeniceus* | 4 |  |  |  |  |  |  |  |  |  |  |  |  |  |  |  |
|  | 1. *Aimophila rufescens* | 1 |  |  | 1 |  |  |  |  |  |  |  |  |  |  |  |  |
|  | 1. *Amazilia yucatanensis* |  |  | 1 | 3 | 5 |  |  |  |  | 3 | 2 |  |  |  |  |  |
|  | 1. *Amazona albifrons* |  |  |  | 6 | 12 |  | 22 |  |  | 10 |  |  |  |  |  |  |
|  | 1. *Amazona autumnalis* | 109 |  |  | 4 | 4 |  |  |  | 4 | 16 | 18 | 10 |  | 12 |  |  |
|  | 1. *Amazona oratrix* |  |  |  |  |  |  |  |  | 2 |  |  |  |  |  |  |  |
|  | 1. *Amazona* sp. |  |  |  |  |  |  |  |  |  |  | 2 |  |  |  |  |  |
|  | 1. *Aramides albiventris* | 2 |  |  | 1 |  |  |  |  |  |  |  |  |  |  |  |  |
|  | 1. *Archilochus colubris* |  |  |  | 4 | 1 |  |  |  | 1 | 2 |  |  |  |  |  |  |
|  | 1. *Ardea alba* | 3 |  |  | 3 | 4 |  |  |  |  |  |  |  |  |  |  |  |
|  | 1. *Atlapetes pileatus* |  |  |  |  |  |  |  |  |  |  |  |  |  |  | 2 |  |
|  | 1. *Aulacorhynchus prasinus* |  |  |  |  |  | 2 |  |  |  |  |  |  |  | 2 |  |  |
|  | 1. *Baeolophus atricristatus* |  |  |  |  |  |  |  |  |  |  |  | 2 |  |  |  |  |
|  | 1. *Basileuterus culicivorus* |  |  |  |  |  |  |  |  |  |  |  |  |  | 2 |  |  |
|  | 1. *Basileuterus rufifrons* |  |  |  |  |  |  |  |  |  |  | 1 |  |  |  |  |  |
|  | 1. *Basilinna leucotis* |  |  |  |  |  |  |  |  |  |  |  |  |  |  | 1 |  |
|  | 1. *Bubulcus ibis* | 4 |  | 2 | 9 |  |  |  |  |  |  |  |  |  |  |  |  |
|  | 1. *Buteo brachyurus* |  |  |  |  |  |  |  |  |  |  | 3 |  |  |  |  |  |
|  | 1. *Buteo jamaicensis* |  |  |  |  |  | 1 |  |  |  |  |  |  |  |  | 2 |  |
|  | 1. *Buteo plagiatus* | 2 |  |  |  |  |  | 2 |  |  | 4 | 5 | 1 |  |  |  |  |
|  | 1. *Buteo platypterus* |  |  |  |  |  |  |  |  | 80 |  |  | 2 |  |  |  |  |
|  | 1. *Buteo swainsoni* |  |  |  |  |  |  |  |  | 10 |  |  |  |  |  |  |  |
|  | 1. *Buteogallus anthracinus* |  |  |  |  |  |  |  |  | 1 |  |  |  |  |  |  |  |
|  | 1. *Butorides virescens* | 1 |  |  | 9 |  | 4 |  |  |  |  |  |  | 4 |  |  |  |
|  | 1. *Campylorhynchus rufinucha* |  |  |  |  | 19 |  | 8 |  |  | 5 | 4 | 10 |  |  |  |  |
|  | 1. *Campylorhynchus zonatus* | 12 | 2 |  | 13 | 12 |  | 10 | 1 | 5 | 8 | 11 | 14 |  | 1 |  |  |
|  | 1. *Caracara plancus* |  |  | 1 | 1 |  |  |  |  |  | 2 | 1 |  |  |  |  |  |
|  | 1. *Cardellina pusilla* |  | 1 | 1 | 4 | 7 | 4 | 4 | 1 | 1 | 3 |  |  | 2 | 2 | 4 |  |
|  | 1. *Cathartes aura* | 3 | 4 | 4 | 15 | 13 | 5 | 4 | 4 | 12 | 7 | 8 |  |  | 2 |  |  |
|  | 1. *Catharus occidentalis* |  |  |  |  |  |  |  |  |  |  |  |  |  |  | 1 |  |
|  | 1. *Catharus ustulatus* |  |  |  |  |  |  |  |  |  |  |  |  |  |  | 1 |  |
|  | 1. *Catherpes mexicanus* | 2 |  |  |  | 13 | 2 |  | 1 |  | 1 | 1 | 7 |  | 1 |  |  |
|  | 1. *Chaetura vauxi* |  |  | 22 | 11 |  |  |  |  |  |  | 270 |  |  |  |  |  |
|  | 1. *Chloroceryle amazona* |  |  |  |  |  |  |  |  |  |  |  |  |  | 1 |  |  |
|  | 1. *Chloroceryle americana* | 6 |  |  | 1 |  |  |  |  |  |  | 1 |  |  |  |  |  |
|  | 1. *Chlorophonia elegantissima* |  |  |  |  |  |  |  |  |  |  | 1 |  |  |  |  |  |
|  | 1. *Chlorospingus flavopectus* |  |  |  |  |  | 3 |  |  |  |  |  |  |  | 2 |  |  |
|  | 1. *Chlorostilbon canivetii* |  |  |  |  |  |  |  |  |  | 1 | 2 |  |  |  |  |  |
|  | 1. *Ciccaba virgata* | 5 |  |  | 1 | 4 | 3 |  |  | 5 | 3 | 2 | 3 | 3 | 1 |  |  |
|  | 1. *Cinclus mexicanus* |  |  |  |  |  |  |  | 1 |  |  |  |  |  |  |  |  |
|  | 1. *Columbina inca* | 8 |  | 4 | 7 |  |  | 18 |  | 2 | 5 | 8 |  | 2 |  | 6 | 2 |
|  | 1. *Columbina passerina* |  |  |  |  | 3 |  |  |  |  |  | 2 | 3 |  |  |  |  |
|  | 1. *Columbina talpacoti* | 4 |  |  | 2 |  |  | 5 |  | 1 |  |  | 2 |  |  |  |  |
|  | 1. *Contopus cooperi* |  |  |  |  |  |  |  |  |  |  |  |  |  |  | 1 |  |
|  | 1. *Contopus pertinax* |  |  |  |  |  |  |  |  |  |  |  |  |  | 1 |  |  |
|  | 1. *Contopus* sp. |  |  |  |  |  | 2 |  |  |  |  |  |  |  |  |  |  |
|  | 1. *Contopus virens* |  |  |  |  |  |  |  |  |  |  |  | 1 |  | 1 |  |  |
|  | 1. *Coragyps atratus* | 29 | 17 | 72 | 144 | 187 | 29 | 41 | 12 | 542 | 48 | 70 | 92 |  | 15 |  |  |
|  | 1. *Cozzyzus americanus* |  |  |  |  |  |  |  |  |  |  |  |  |  |  |  | 1 |
|  | 1. *Crotophaga sulcirostris* | 1 |  |  | 3 | 8 |  | 17 |  | 2 | 3 | 4 | 2 |  |  |  |  |
|  | 1. *Crypturellus cinnamomeus* | 3 |  |  |  |  |  |  |  |  | 1 | 3 |  |  |  |  |  |
|  | 1. *Cyanerpes cyaneus* |  |  |  |  |  |  |  |  | 1 |  |  |  |  |  |  |  |
|  | 1. *Cyanocitta stelleri* |  |  |  |  |  |  |  |  |  |  |  |  |  |  | 14 | 6 |
|  | 1. *Cyanocompsa parellina* |  |  |  |  | 3 |  |  |  |  |  |  |  |  |  |  |  |
|  | 1. *Dendrocygna autumnalis* | 4 |  |  |  |  |  |  |  |  |  |  | 4 |  |  |  |  |
|  | 1. *Dives dives* | 14 |  | 4 |  |  |  | 29 | 9 | 12 | 4 | 2 | 29 |  |  |  |  |
|  | 1. *Dryobates scalaris* | 1 |  |  |  |  |  |  |  |  |  |  |  |  |  |  |  |
|  | 1. *Dryobates villosus* |  |  |  |  |  |  |  |  |  |  |  |  |  |  | 1 |  |
|  | 1. *Dryocopus lineatus* | 1 |  |  |  |  |  |  |  | 3 |  |  | 1 | 2 |  |  |  |
|  | 1. *Dumetella carolinensis* |  |  |  | 3 | 2 |  |  |  | 5 |  |  | 1 |  |  |  |  |
|  | 1. *Elanus leucurus* |  |  |  |  |  |  |  |  |  |  |  |  |  |  |  | 2 |
|  | 1. *Empidonax minimus* |  |  |  | 1 |  |  |  |  |  |  |  |  |  | 1 |  |  |
|  | 1. *Empidonax occidentalis* |  |  |  | 1 |  |  |  |  | 2 |  | 1 |  |  |  |  |  |
|  | 1. *Empidonax* sp. |  |  |  |  |  | 3 |  |  | 1 | 1 | 1 |  | 1 |  | 2 | 1 |
|  | 1. *Eudocimus albus* |  |  |  |  |  | 1 |  |  |  |  |  |  |  |  |  |  |
|  | 1. *Euphonia affinis* |  |  |  |  |  |  |  |  | 1 |  |  |  |  |  |  |  |
|  | 1. *Euphonia hirundinacea* |  |  |  |  | 5 |  |  |  | 2 |  | 2 |  |  |  |  |  |
|  | 1. *Falco columbarius* |  |  |  |  |  |  |  |  |  | 2 |  |  |  |  |  |  |
|  | 1. *Falco femoralis* |  |  |  |  | 3 |  |  |  |  |  |  |  |  |  |  |  |
|  | 1. *Falco peregrinus* |  |  |  |  |  |  |  |  | 1 |  |  |  |  |  |  |  |
|  | 1. *Falco rufigularis* |  |  | 2 |  | 3 |  |  |  | 1 |  |  | 1 |  | 1 |  |  |
|  | 1. *Geothlypis* sp. |  |  |  |  |  |  |  |  | 1 |  |  |  |  |  |  |  |
|  | 1. *Geothlypis tolmiei* | 1 |  |  |  |  |  |  |  |  |  |  |  |  |  | 1 |  |
|  | 1. *Geothypis trichas* |  |  |  |  |  |  |  |  |  | 1 |  |  |  |  | 1 |  |
|  | 1. *Glaucidium brasilianum* | 5 |  | 3 | 4 | 2 | 3 | 4 | 2 | 2 | 6 | 7 | 4 | 2 | 1 |  | 1 |
|  | 1. *Haeomorhous mexicanus* |  |  |  |  |  |  |  |  |  |  |  | 1 |  |  | 5 | 1 |
|  | 1. *Herpetoteres cachinnans* | 1 |  | 1 |  |  |  |  |  |  | 2 |  | 1 |  | 1 |  |  |
|  | 1. *Hilorchylus sumichrasti* | 2 |  |  |  |  |  |  |  |  |  |  |  |  |  |  |  |
|  | 1. *Hirundo rustica* |  |  |  |  |  |  |  |  |  |  |  | 15 |  |  |  | 5 |
|  | 1. *Icteria virens* |  |  |  |  |  |  |  |  |  |  |  | 2 |  |  |  |  |
|  | 1. *Icterus bullockii* |  |  |  |  |  |  |  |  |  |  |  |  |  |  | 1 |  |
|  | 1. *Icterus galbula* | 2 |  |  |  |  |  | 4 |  | 3 | 2 | 2 |  |  |  |  |  |
|  | 1. *Icterus gularis* | 1 |  | 2 |  | 4 |  | 6 | 6 | 1 | 2 | 4 | 2 | 1 |  |  |  |
|  | 1. *Junco phaeonotus* |  |  |  |  |  |  |  |  |  |  |  |  |  |  | 13 | 9 |
|  | 1. *Lampornis clemenciae* |  |  |  |  |  |  |  |  |  |  |  |  | 1 |  |  | 4 |
|  | 1. *Leiothlypis celata* |  |  |  |  |  |  |  |  | 1 |  |  |  |  |  |  |  |
|  | 1. *Leiothlypis ruficapilla* | 1 |  |  |  |  |  |  |  |  |  |  |  |  | 1 |  |  |
|  | 1. *Leptotila verreauxi* | 5 | 1 | 7 | 3 | 9 | 2 | 5 | 3 | 2 | 2 | 6 | 8 |  |  |  |  |
|  | 1. *Lonchura malacca* | 9 |  |  |  |  |  |  |  |  |  |  |  |  |  |  |  |
|  | 1. *Loxia curvirostra* |  |  |  |  |  |  |  |  |  |  |  |  |  |  | 5 |  |
|  | 1. *Megarynchus pitangua* | 3 |  | 2 | 5 |  | 4 |  | 2 | 1 | 3 | 12 | 6 |  |  |  |  |
|  | 1. *Melanerpes arifrons* | 7 |  | 3 | 9 | 13 | 1 | 5 | 1 | 9 | 4 | 10 | 11 | 2 | 2 |  |  |
|  | 1. *Melanerpes formicivorus* |  |  |  |  |  |  |  |  |  | 4 | 3 | 6 |  | 1 | 2 |  |
|  | 1. *Melozpisa lincolnii* |  |  |  | 1 |  |  |  |  |  |  |  |  |  |  |  |  |
|  | 1. *Micrastur semitorquatus* |  |  |  |  |  |  |  |  |  |  |  | 1 |  |  |  |  |
|  | 1. *Mitrephanes phaeocercus* |  |  |  |  |  |  |  |  |  |  |  |  |  | 2 |  |  |
|  | 1. *Mniotilta varia* |  |  | 2 | 1 |  |  |  |  |  | 4 |  |  |  | 4 |  |  |
|  | 1. *Molothrus aeneus* | 8 |  | 1 |  |  |  | 5 |  | 3 |  | 1 | 2 |  |  | 1 |  |
|  | 1. *Momotus coeruliceps* |  |  |  |  |  |  |  |  |  |  | 3 | 1 | 1 |  |  |  |
|  | 1. *Momotus lessonii* | 3 |  |  | 3 |  |  |  |  |  |  |  | 3 |  |  |  |  |
|  | 1. *Momotus* sp. | 2 |  |  |  |  |  |  |  | 1 |  |  |  |  |  |  |  |
|  | 1. *Myadestes occidentalis* |  |  |  |  |  | 2 |  |  | 1 |  |  |  |  | 6 | 6 | 3 |
|  | 1. *Myadestes unicolor* | 3 |  |  |  |  |  |  |  |  |  |  |  |  |  |  |  |
|  | 1. *Myarchus tuberculifer* | 2 | 1 |  | 2 |  |  |  |  |  |  | 1 | 4 |  | 1 |  |  |
|  | 1. *Myiozetetes similis* | 6 |  | 6 | 6 | 7 |  | 13 |  | 3 | 13 | 13 | 7 |  |  |  |  |
|  | 1. *Myoborus miniatus* |  |  |  |  |  |  |  |  |  |  |  |  |  |  | 8 | 2 |
|  | 1. *Myodynastes luteiventris* | 1 |  |  |  | 4 |  |  |  |  |  | 2 | 1 |  |  |  |  |
|  | 1. *Nannopterum brasilianum* |  |  |  | 42 |  |  |  |  |  |  |  |  |  |  |  |  |
|  | 1. *Nyctibius jamaicensis* |  |  |  |  |  |  |  |  |  |  | 1 |  |  |  |  |  |
|  | 1. *Nyctidromus albicollis* |  |  |  |  | 4 |  |  |  |  |  |  | 1 |  |  |  |  |
|  | 1. *Oreothlypis superciliosa* |  |  |  |  |  |  |  |  |  |  |  |  |  |  | 4 |  |
|  | 1. *Ortalis vetula* | 6 |  |  | 1 | 2 |  | 3 | 1 | 7 | 4 | 2 | 4 | 2 |  |  |  |
|  | 1. *Pachyramphus aglaiae* |  |  |  |  |  |  |  |  |  |  |  | 1 |  |  |  |  |
|  | 1. *Pampa curvipennis* |  |  |  |  | 5 |  |  |  |  |  |  |  |  |  |  |  |
|  | 1. *Parkesia motacilla* |  |  |  | 1 |  |  |  |  |  |  |  | 4 |  |  |  |  |
|  | 1. *Parkesia noveboracensis* |  |  |  |  |  |  |  | 1 |  |  |  |  |  |  |  |  |
|  | 1. *Passer domesticus* |  |  |  |  | 2 |  |  |  | 1 |  |  |  |  |  | 6 |  |
|  | 1. *Passerina caerulea* |  |  |  |  | 13 |  |  |  |  |  |  |  |  |  |  |  |
|  | 1. *Passerina cyanea* | 6 |  |  |  |  |  | 2 |  |  |  |  | 3 |  | 1 |  |  |
|  | 1. *Passerina versicolor* |  |  |  |  |  |  |  |  |  |  |  | 1 |  |  |  |  |
|  | 1. *Patagioenas flavirostris* | 9 | 2 | 4 | 7 |  |  | 5 | 2 | 2 | 3 | 8 | 7 |  |  |  |  |
|  | 1. *Peucedramus taeniatus* |  |  |  |  |  |  |  |  |  |  |  |  |  |  |  | 1 |
|  | 1. *Peucthicus ludovicianus* | 1 |  |  |  |  |  |  |  | 1 |  |  |  |  |  |  |  |
|  | 1. *Peucthicus melanocephalus* |  |  |  |  |  |  |  |  |  |  |  |  |  |  | 2 |  |
|  | 1. *Phaethornis striigularis* |  |  | 1 | 1 |  |  |  |  |  | 2 | 1 | 1 |  |  |  |  |
|  | 1. *Pheugopedius maculipectus* |  |  | 5 | 2 |  |  | 1 |  |  | 1 |  | 1 |  |  |  |  |
|  | 1. *Piaya cayana* |  |  | 1 |  |  | 1 |  |  |  | 1 |  | 2 |  | 1 |  |  |
|  | 1. *Pionus senilis* | 30 |  | 3 |  |  | 30 |  | 30 | 12 |  |  |  |  |  |  |  |
|  | 1. *Pipilo ocai* |  |  |  |  |  |  |  |  |  |  |  |  |  |  | 2 |  |
|  | 1. *Piranga bidentata* |  |  |  |  |  |  |  |  | 1 |  |  |  |  | 3 |  |  |
|  | 1. *Piranga rubra* |  |  |  |  |  |  |  | 1 | 5 |  |  |  |  | 1 |  |  |
|  | 1. *Pitangus sulphuratus* | 1 |  |  | 4 |  |  | 3 | 1 |  | 2 | 2 | 5 |  |  |  |  |
|  | 1. *Poecile sclateri* |  |  |  |  |  |  |  |  |  |  |  |  |  |  | 2 | 5 |
|  | 1. *Polioptilia caerulea* | 4 |  | 3 |  | 8 |  | 5 |  | 3 | 5 | 2 | 6 | 2 | 6 | 3 |  |
|  | 1. *Psaltriparus minimus* |  |  |  |  |  |  |  |  |  |  |  |  |  |  | 6 | 5 |
|  | 1. *Psarocolius montezuma* | 11 |  | 7 | 10 | 10 | 16 | 19 | 22 | 89 | 11 | 26 | 16 | 2 | 2 |  |  |
|  | 1. *Psarocolius wagleri* |  |  |  |  |  |  |  |  |  |  |  |  | 1 |  |  |  |
|  | 1. *Psilorhinus morio* | 11 |  | 13 | 4 | 30 | 5 | 7 |  | 7 | 2 | 8 | 10 | 9 | 7 |  |  |
|  | 1. *Ptiliogonys cinereus* |  |  |  |  |  |  |  |  |  |  |  |  |  |  | 9 | 4 |
|  | 1. *Pyrocephalus rubinus* |  |  |  | 1 |  |  |  |  |  |  |  |  |  |  |  |  |
|  | 1. *Quiscalus mexicanus* |  |  | 8 |  | 5 |  |  |  |  | 2 | 12 |  |  |  | 4 |  |
|  | 1. *Ramphastos sulfuratus* |  |  |  |  |  |  |  |  |  |  |  | 3 |  |  |  |  |
|  | 1. *Ramphastos sulfuratus* | 3 |  | 1 | 1 | 4 |  |  |  | 6 | 5 | 6 | 2 |  |  |  |  |
|  | 1. *Rodothraupis celaeno* |  |  |  |  | 3 |  |  |  |  |  |  |  |  | 1 |  |  |
|  | 1. *Rupornis magnirostris* | 3 | 1 |  | 5 | 8 |  | 2 |  | 4 | 1 | 3 | 1 | 3 | 1 | 1 | 1 |
|  | 1. *Saltator atriceps* | 9 |  | 7 | 4 |  |  | 5 |  | 13 | 2 | 5 | 12 |  |  |  |  |
|  | 1. *Saltator coerulescens* |  |  |  | 2 |  |  |  |  |  |  |  |  |  |  |  |  |
|  | 1. *Saucerottia beryllina* |  |  |  |  |  |  |  |  |  |  |  |  | 1 | 3 |  |  |
|  | 1. *Saucerottia cyanocephala* |  |  |  |  |  |  |  | 1 | 2 | 2 | 1 |  | 1 |  |  |  |
|  | 1. *Sayornis nigricans* | 3 | 1 | 1 | 1 | 3 | 2 |  | 2 |  |  |  |  |  | 1 |  |  |
|  | 1. *Sayornis phoebe* |  |  |  | 1 |  |  |  |  |  |  |  | 1 |  |  |  |  |
|  | 1. *Setophaga citrina* |  |  |  |  |  |  |  |  |  |  |  |  |  | 1 |  |  |
|  | 1. *Setophaga coronata* |  |  |  |  |  |  |  |  | 3 | 2 |  |  |  |  |  |  |
|  | 1. *Setophaga magnolia* |  |  |  | 1 |  |  |  |  |  | 2 |  | 2 |  | 1 |  |  |
|  | 1. *Setophaga ruticilla* |  |  | 1 |  | 7 | 1 |  |  |  |  |  |  |  | 2 | 1 |  |
|  | 1. *Setophaga virens* |  |  | 2 | 2 | 3 |  | 3 | 1 | 2 | 4 |  | 5 | 3 | 4 | 2 |  |
|  | 1. *Sittasomus griseicapillus* | 3 |  |  |  |  |  |  |  |  |  |  |  |  | 7 |  |  |
|  | 1. *Spinus pinus* |  |  |  |  |  |  |  |  |  |  |  |  |  |  |  | 2 |
|  | 1. *Spinus pitiayumi* |  |  |  |  |  |  |  |  |  |  |  | 1 |  |  |  |  |
|  | 1. *Spinus psaltria* | 1 |  |  | 4 |  |  | 3 |  | 1 |  | 1 |  |  |  | 1 |  |
|  | 1. *Spizella pallida* |  |  |  |  |  |  |  |  |  |  |  |  |  |  | 1 |  |
|  | 1. *Spizella passerina* |  |  |  |  |  |  |  |  |  |  |  |  |  |  | 2 |  |
|  | 1. *Sporophila morelleti* | 6 |  |  | 6 |  |  |  |  |  | 4 | 3 | 2 |  |  |  |  |
|  | 1. *Stelgidopteryx serripennis* | 160 | 6 | 14 | 54 | 164 | 32 | 78 | 12 |  |  |  | 2 |  |  | 13 | 71 |
|  | 1. *Streptopelia decaocto* |  |  |  | 9 | 2 |  |  |  |  |  |  | 1 |  |  | 3 |  |
|  | 1. *Streptoprocne zonaris* |  |  |  |  | 11 |  |  |  |  |  | 34 |  |  |  |  |  |
|  | 1. *Tachybaptus dominicus* |  |  |  |  |  |  |  |  |  |  |  | 2 |  |  |  |  |
|  | 1. *Tachycineta thalassina* |  |  |  |  |  |  |  |  |  |  |  |  |  |  | 16 |  |
|  | 1. *Thamnophilus doliatus* |  |  |  |  |  |  |  | 2 | 1 |  |  |  |  |  |  |  |
|  | 1. *Thraupis episcopus* |  |  |  |  | 4 |  | 2 |  | 3 | 2 | 3 | 3 |  | 1 |  |  |
|  | 1. *Thryomanes bewickii* |  |  |  |  |  |  |  | 1 |  |  |  |  |  |  |  |  |
|  | 1. *Tiaris olivaceus* |  |  |  |  |  |  |  |  |  |  | 1 | 3 |  |  |  |  |
|  | 1. *Tityra semifasciata* | 2 |  |  | 9 |  |  |  |  | 10 | 6 | 7 | 12 |  |  |  |  |
|  | 1. *Tolmomyias sulphurescens* |  |  |  |  |  |  |  |  | 1 |  |  |  |  |  |  |  |
|  | 1. *Trauphis abbas* | 3 |  |  | 3 | 2 |  | 1 |  | 9 | 7 | 7 | 3 |  |  |  |  |
|  | 1. *Troglodites aedon* | 2 |  | 1 | 2 |  |  | 1 |  |  |  |  |  |  | 5 |  |  |
|  | 1. *Trogon caligatus* |  |  |  |  |  |  |  |  | 2 |  | 1 |  |  | 1 |  |  |
|  | 1. *Turdus assimilis* |  |  |  |  |  |  |  |  |  |  |  |  |  | 3 |  |  |
|  | 1. *Turdus grayi* | 4 |  | 2 | 5 | 4 |  | 5 |  | 3 | 2 | 2 | 5 |  |  | 1 | 3 |
|  | 1. *Turdus migratorius* |  |  |  |  |  |  |  |  |  |  |  |  |  |  | 11 | 4 |
|  | 1. *Turdus* sp. |  |  |  |  |  |  |  |  |  |  |  |  |  | 1 |  |  |
|  | 1. *Tyrannus forficatus* |  |  |  |  |  |  |  |  |  |  | 2 |  |  |  |  |  |
|  | 1. *Tyrannus melancholicus* | 6 |  |  | 1 |  |  |  |  | 1 |  | 3 | 7 |  |  |  |  |
|  | 1. *Tyto alba* | 2 |  | 1 |  | 4 | 2 | 2 | 1 | 1 | 2 |  |  |  | 3 |  | 2 |
|  | 1. *Uropsila leucogastra* |  |  |  |  |  |  |  |  | 1 | 2 | 2 |  | 1 |  |  |  |
|  | 1. *Vireo griseus* |  |  | 1 |  | 2 |  |  |  | 2 |  |  |  |  | 2 |  |  |
|  | 1. *Vireo solitarius* |  |  | 1 | 1 |  |  |  |  |  |  |  |  |  | 2 |  |  |
|  | 1. *Volatinia jacarina* | 8 |  |  | 3 | 4 |  |  |  |  | 2 |  | 2 |  |  |  |  |
|  | 1. *Zenaida asiatica* | 1 |  |  | 1 |  |  | 5 |  | 1 |  | 1 |  |  |  | 4 |  |
|  | 1. *Sin ID* | 3 |  | 5 | 4 |  | 8 | 2 | 2 | 5 | 2 | 3 | 6 | 4 | 3 | 6 | 2 |
| Mammalia | 1. *Artibeus jamaicensis* |  |  |  | 132 |  | 60 |  | 25 | 369 | 370 | 30 | 965 |  |  |  |  |
|  | 1. *Balantioteryx io* |  |  | 17 | 95 |  |  |  |  |  |  |  |  |  |  |  |  |
|  | 1. *Balantioteryx plicata* |  |  | 15 | 35 |  |  |  |  |  |  |  |  |  |  |  |  |
|  | 1. *Bassariscus astutus* |  |  |  |  |  |  |  |  |  |  |  |  |  |  | 1 |  |
|  | 1. *Canis familiaris* |  |  |  |  | 1 |  |  |  |  |  |  |  |  |  | 1 |  |
|  | 1. *Carollia sowelli* |  |  |  |  |  | 2 |  |  |  |  |  |  |  |  |  |  |
|  | 1. *Carollia sp.* |  |  |  |  |  |  |  |  | 11 | 25 | 55 | 12 |  |  |  |  |
|  | 1. *Corynorhinus sp.* |  |  |  |  |  |  |  |  |  |  |  |  |  |  |  | 43 |
|  | 1. *Corynorhynus mexicanus* |  |  |  |  |  |  |  |  |  |  |  |  |  |  | 5 | 63 |
|  | 1. *Cuniculus paca* |  |  |  |  |  |  |  | 2 |  |  |  |  |  |  |  |  |
|  | 1. *Desmodus rotundus* |  |  |  |  | 225 |  |  |  |  | 45 | 5 |  | 20 |  |  |  |
|  | 1. *Didelphis* sp. |  |  |  |  | 1 | 2 |  | 1 | 1 |  |  |  | 1 |  |  |  |
|  | 1. *Diphylla ecaudata* |  |  |  |  |  | 1 | 2 |  |  | 35 |  | 7 | 25 |  |  |  |
|  | 1. *Felis catus* |  |  |  |  |  |  | 1 |  |  |  |  |  |  |  |  |  |
|  | 1. *Glossophaga* sp. |  |  | 15 |  |  |  |  |  | 5 |  | 15 |  |  |  |  |  |
|  | 1. *Leptonycteris yerbabuenae* |  |  |  |  |  |  |  |  |  |  | 5 |  |  |  |  |  |
|  | 1. *Mimon cozumelae* |  |  |  |  |  |  |  |  | 6 |  |  |  |  |  |  |  |
|  | 1. *Miotys keaysi* |  |  |  |  |  |  |  | 2950 |  |  |  |  |  | 8 |  |  |
|  | 1. *Miotys velifer* |  |  |  |  |  |  |  | 320 |  |  |  |  |  |  |  |  |
|  | 1. *Mormoops megalophylla* |  |  |  | 5 |  |  |  |  |  |  |  |  |  |  |  |  |
|  | 1. *Myotis* sp. |  | 100 |  | 10 |  |  |  | 550 |  |  |  |  |  | 25 | 745 | 170 |
|  | 1. *Nasua narica* |  |  |  |  |  | 1 |  |  |  |  |  |  |  |  |  |  |
|  | 1. *Natalus mexicanus* |  |  |  |  |  |  |  | 20 |  |  | 17 |  |  |  |  |  |
|  | 1. *Neotoma* sp. |  |  |  |  |  |  |  |  |  |  |  |  |  |  |  | 1 |
|  | 1. *Perimyotis subflavus* |  |  |  |  |  |  |  |  |  |  |  |  |  |  |  | 6 |
|  | 1. *Peromyscus furvus* |  |  |  |  |  |  |  |  |  |  |  | 1 |  |  |  |  |
|  | 1. *Peromyscus mexicanus* |  |  |  |  |  |  |  |  | 1 |  |  |  |  |  |  |  |
|  | 1. *Peromyscus* sp. |  |  |  |  |  |  |  |  |  |  |  | 1 |  |  |  |  |
|  | 1. *Procyon lotor* |  |  |  |  |  |  |  |  |  | 1 | 1 |  |  |  |  |  |
|  | 1. *Tamandua mexicana* | 1 |  |  |  |  |  |  |  |  |  |  |  |  |  |  |  |
|  | 1. *Tylomys nudicaudus* |  |  |  |  |  |  |  |  |  |  | 1 |  |  |  |  |  |
|  |  |  |  |  |  |  |  |  |  |  |  |  |  |  |  |  |  |
|  |  |  |  |  |  |  |  |  |  |  |  |  |  |  |  |  |  |

Z1: Ojo de agua grande cave (OJ), Nacimiento de 7 aguas cave (NC), Atoyac cave (AY) maguey cave (MY)

Z2: Tenampa cave (TN), Pintada cave (PI), Tepeapulco basement (TP), boqueron de Capulapa cave (BQ), la Palma cave (PL), el Aire cave (AI), la Reja cave (RE) Pinoltepec cave (PN).

Z3: La Garganta cave (GT), Maltos basement (MT).

Z4: La Escalera cave (ES) Volcancillo cave (VL).
